# CRLNet: A Multimodal Peach Detection Network Based on Cooperative Asymptotic Enhancement and the Fusion of Granularity Refinement

**DOI:** 10.3390/plants13141980

**Published:** 2024-07-19

**Authors:** Jiahao Liu, Chaoying He, Mingfang Wang, Yichu Jiang, Manman Sun, Miying Yan, Mingfang He

**Affiliations:** 1 College of Electronic Information and Physics, Central South University of Forestry and Technology, Changsha 410004, China; hao@csuft.edu.cn (J.L.); t20040528@csuft.edu.cn (C.H.); t20060566@csuft.edu.cn (M.W.); 2 Hunan Polytechnic of Environment and Biology, Hengyang 421005, China; jiangyichu@hnebp.edu.cn; 3 Bangor College China, Central South University of Forestry and Technology, Changsha 410004, China; mns21rty@bangor.ac.uk

**Keywords:** deep learning, granularity refinement, intelligent agriculture, multimodal detection, peach detection models

## Abstract

Accurate peach detection is essential for automated agronomic management, such as mechanical peach harvesting. However, ubiquitous occlusion makes identifying peaches from complex backgrounds extremely challenging. In addition, it is difficult to capture fine-grained peach features from a single RGB image, which can suffer from light and noise in scenarios with dense small target clusters and extreme light. To solve these problems, this study proposes a multimodal detector, called CRLNet, based on RGB and depth images. First, YOLOv9 was extended to design a backbone network that can extract RGB and depth features in parallel from an image. Second, to address the problem of information fusion bias, the Rough–Fine Hybrid Attention Fusion Module (RFAM) was designed to combine the advantageous information of different modes while suppressing the hollow noise at the edge of the peach. Finally, a Transformer-based Local–Global Joint Enhancement Module (LGEM) was developed to jointly enhance the local and global features of peaches using information from different modalities in order to enhance the percentage of information about the target peaches and remove the interference of redundant background information. CRLNet was trained on the Peach dataset and evaluated against other state-of-the-art methods; the model achieved an $mAP_{50}$ of 97.1%. In addition, CRLNet also achieved an $mAP_{50}$ of 92.4% in generalized experiments, validating its strong generalization capability. These results provide valuable insights for peach and other outdoor fruit multimodal detection.

## 1. Introduction

Peach is a tropical fruit grown in large quantities and is an excellent source of vitamins and sugar [1]. At present, peach picking relies on manual labor, which has a low efficiency and high cost [2]. The use of machine vision technology to achieve automatic peach picking using a machine can reduce labor costs and increase productivity [3,4,5,6]. Due to the unstructured growing environment of peaches, the image content acquired by the picking robot is varied and complex; for example, under the shade of branches and changing light [7]. Hence, the accurate recognition and localization of peaches are the key factors enabling the robot to complete the task [8,9]. Therefore, excellent visual detection algorithms [10,11] are crucial.

Methods based on traditional image processing techniques have been widely used in peach detection tasks [12,13]. These methods mainly extract image features through the manual formation of rules and the use of decision-level fusion of multiple classifiers to achieve the localization and classification of different kinds of peaches [14]. However, these methods are highly susceptible to environmental changes and have shortcomings such as a poor generalization ability and robustness. In recent years, deep learning algorithms have developed rapidly in the field of image processing [15]. Many researchers have chosen to use convolutional neural networks (CNNs) to achieve peach detection [9,16,17,18]. Among the CNN-based methods [19,20,21], the You Only Look Once (YOLO) [8,22,23,24] series works best. YOLOv3 is the first end-to-end real-time detection model better than the contemporaneous detection algorithms, such as Fastest-Rcnn [25], Single Shot MultiBox Detector (SSD) [26], and so on. In contrast to the two-stage detection algorithm, YOLOv3 uses a network called Darknet53 for feature extraction, then proceeds to multi-scale fusion of the extracted features, and, finally, the detector head decouples the multi-scale features to classify and localize the object. On the basis of this, YOLOv5 [8] introduced the C3 module with rich information flow and the SPPF [27] module that performs multi-scale feature fusion to further improve the performance of the network. To allow the network to be more flexible, YOLOv8 [23] uses an anchorless detection header and no longer needs to generate pre-selected boxes via clustering. YOLOv9 [24] uses a backbone called GELAN for feature extraction; the network has powerful feature extraction capabilities while maintaining lightness and speed, and GELAN provides support for Programmable Gradient Information (PGI), which improves the detection accuracy.

However, there are many challenging tasks when detecting peaches in real-world environments, such as obscuration, overlap, and constantly changing light; therefore, using only an RGB camera as a source of data is not desirable [28]. With the development of sensor technology, more information (e.g., depth and Inf) provides us with new perspectives to solve the problem [29]. Qing et al. [30] spliced the RGB peach image, depth peach image, and infrared peach image to obtain a five-channel fused image and used the YOLOv5s detector to detect the fused image, and they achieved an $mAP_{50}$ of 88.9% for bagged peach detection. Similarly, Nguyen et al. [31] processed RGB images using depth images and then used the processed images for detection. However, these input-level multimodal detections only fused the multimodal information at the pixel level, neglecting the processing of high-level semantic information. To solve this problem, many researchers have attempted the feature-level fusion of multimodal information to break the barrier between the highly different types of information. Sun et al. [32] proposed a noise-tolerant feature fusion network, which performs feature extraction on an RGB image and a depth image, respectively, through a two-stream network, and then designed an attention-based fusion module to achieve the fusion of different modal information while filtering noise. They achieved an $mAP_{50}$ of 93.1% in citrus detection. Some researchers have achieved excellent accuracy while preserving speed through expanding the single-stage detector. Cho et al. [19] used an end-to-end multimodal detector, YOLOv3, for tomato and branching point detection. The backbone of the network was expanded to a dual stream, which allowed for simultaneous feature extraction for RGB and depth. The different modal features were summed and fed into the common neck and head sections, achieving an $mAP_{50}$ of 89.6%. This expansion was the same as in Sharma et al. [33]. As end-to-end detection networks grow, more competitive feature extraction backbones continue to emerge. Wu et al. [34] extended YOLOv7 to dual streams to simultaneously process RGB and depth images and designed a single-direction fusion module based on spatial attention to achieve the accurate detection of tea buds, reaching 91.12% at $mAP_{50}$ with guaranteed real-time performance.

**Figure 1:**
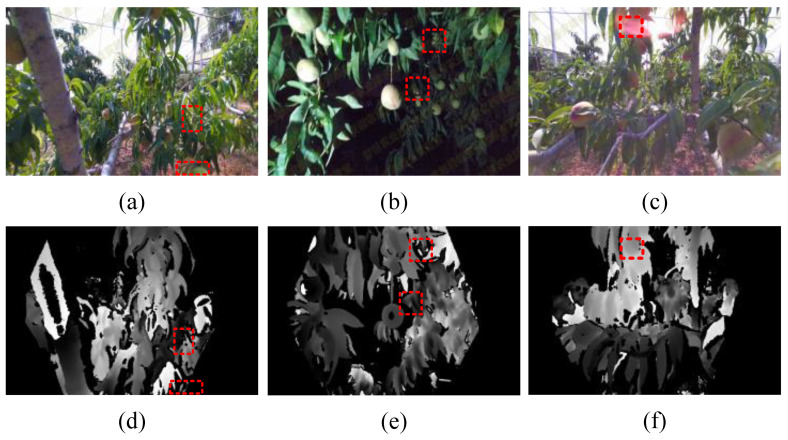
Difficult scenes for peach detection: (**a**) with normal light, (**b**) with dark light at night, and (**c**) with the presence of glare. (**d**–**f**) are depth images of the same scene in (**a**–**c**).

Although existing multimodal detectors have achieved better results in various fields, there are still two problems to be solved in fruit detection tasks, such as those involving peaches: (1) Due to the different imaging principles of various sensors, different types of images have large variation, as shown in Figure 1; hence, simply combining the features of different modalities cannot adequately fuse the high-level semantic information, and the advantages of multimodal information cannot be fully utilized. (2) In a normal lighting environment, as shown in Figure 1a, the information of different modalities is complementary. However, the light in the orchard is uncontrolled, as shown in Figure 1b,c, and there is a large amount of light pollution information in the RGB information in extreme dark light and glare scenarios, and inputting the pollution information into the feature extraction block will introduce too much redundant information into the network, crowding out the favorable information, resulting in the limitation of the model’s performance.

To solve the problem of simple fusion methods not adequately fusing high-level semantic information, Zhang et al. [35] designed a fusion module named FFM, which can fuse RGB and depth information by cross attention; however, there is no further processing after the cross-attention computation, and too many MLP layers also introduce a higher computational burden. Many researchers have also fused information using lightweight attention mechanisms. Fang et al. [36] used spatial attention and channel attention to fuse differential mode features and common mode features, respectively, based on the idea of divide and conquer; however, the attention-based approach is more about micro-tuning the different modal information from the channel or spatial dimensions, and it has limited fusion capability for semantic information. In view of these problems, the present study develops a gradual fusion module called RFAM, which can first perform cross-modal semantic aggregation of RGB and depth using a Transformer; then, it post-processes the aggregated features using a shared MLP layer at a lower cost. Finally, a novel pixel-by-pixel multi-dimensional attention is designed to fine-tune the aggregated features.

To address the issue of the model performance limitations caused by uncontrolled lighting and to enhance the robustness of the model for detection in uncontrolled lighting environments, this study designed a global–local joint enhancement module named LGEM. This module uses depth images, which are robust in complex environments, to restore the RGB images affected by light pollution. To ensure the efficiency of the enhancement module and avoid adding an excessive computational burden while achieving structural enhancement, the module first uses lightweight MLP layers to compress the mixed modal features, obtaining enhancement weights with local information. Then, it employs Transformer layers with global receptive fields to model the structure of the local information weights, which have a channel count of one, thereby restoring the overall structure of the RGB image with minimal computational cost.

Specifically, this research makes the following contributions:

(1) In response to the problem that peach detection cannot achieve higher accuracy using individual RGB images, this study developed a multimodal detection framework called CRLNet, which can simultaneously use RGB images and Depth images to perform peach detection tasks.

(2) In order to achieve a higher detection accuracy, information from different modalities can be fully mixed. This study developed a feature fusion module called RFAM. Firstly, the transformer is used to spatially aggregate multimodal features with significant differences. Then, a cross modal coordinate attention mechanism is used to fine tune the initially aggregated features pixel by pixel, achieving fine-grained fusion of multimodal features.

(3) In order to alleviate the negative impact of uncontrolled lighting on detection tasks in orchards, a feature enhancement module called LGEM was been developed to perform multi-scale restoration on contaminated image features. Firstly, the multimodal features are compressed using the MLP layer to obtain a local enhanced weight map. Then, a self attention mechanism is used to construct a global structural enhanced weight map. Finally, spatial weight maps of different scales are used to repair and enhance the multimodal features.

(4) Compared with seven state-of-the-art single-mode detectors and four state-of-the-art multimodal detectors, CRLNet showed the most competitive performance, $mAP_{50}$ reached 97.1%, Precision reached 94.8%, and Recall reached 88.2%. In addition, CRLNet still performed the best in the detection task of bagged peaches, proving its excellent generalization ability.

## 2. Material and Methods

### 2.1. Acquisition of Datasets

The focus of this paper is peach, with a specific focus on the various stages of growth during the actual orchard production process. Data were obtained from a multimodal peach dataset in an unstructured environment collected by Anhui Agricultural University in 2021 (https://download.scidb.cn/download?fileId=62cc068edd6f884c9c9b9c7d (accessed on 9 July 2022)). The dataset comprises multimodal images of peaches in outdoor unstructured environments at the stages of combing, bagging, and picking. These images were captured using RGB, depth, and infrared modalities. Additionally, images of the fruit were captured at each stage under a range of conditions, including sunny and cloudy days, day and night, with and without bagging, and with or without motion blur. A total of 1000 sets of aligned RGB, depth, and IR images of young peaches were selected from the publicly available multimodal peach dataset. The images were divided into four categories: False, Leaf, Branch, and Fruit. Each category represented a specific type of shading situation: no shading, shading by leaves, shading by branches, and shading by other fruits, respectively.

### 2.2. CRLNet

Existing network designs fail to address the issue of significant information loss during the process of feature extraction in a single layer. The feature extraction network of YOLOv9 is equipped with ELAN blocks, which provide a comprehensive stream of gradient information, enabling the network to perform feature extraction on the input image without loss. In order to enhance the network’s capacity for gradient information, auxiliary information streams are incorporated into the network, thereby conferring a more robust learning ability. In light of the impressive performance exhibited by YOLOv9, it was selected for further investigation in this study. Specifically, CRLNet replicated the backbone used for feature extraction and processed the input RGB and depth images in parallel. In order to allow the network to suppress disturbances such as flare and noise during feature extraction, the LGEM was embedded in the same stage of the backbone. Then, the RGB and depth features were extracted from the dual-stream backbones, respectively. They were fused using the RFAM, followed by inputting the obtained adequately blended features into the ELAN-SPP of YOLOv9 for multi-scale feature fusion and, finally, predicting the detection boxes and categories using the detection head of YOLOv9. For the auxiliary stream part, it was found that the RGB possessed richer information, and the RGB was used as the input to the auxiliary stream.

**Figure 2:**
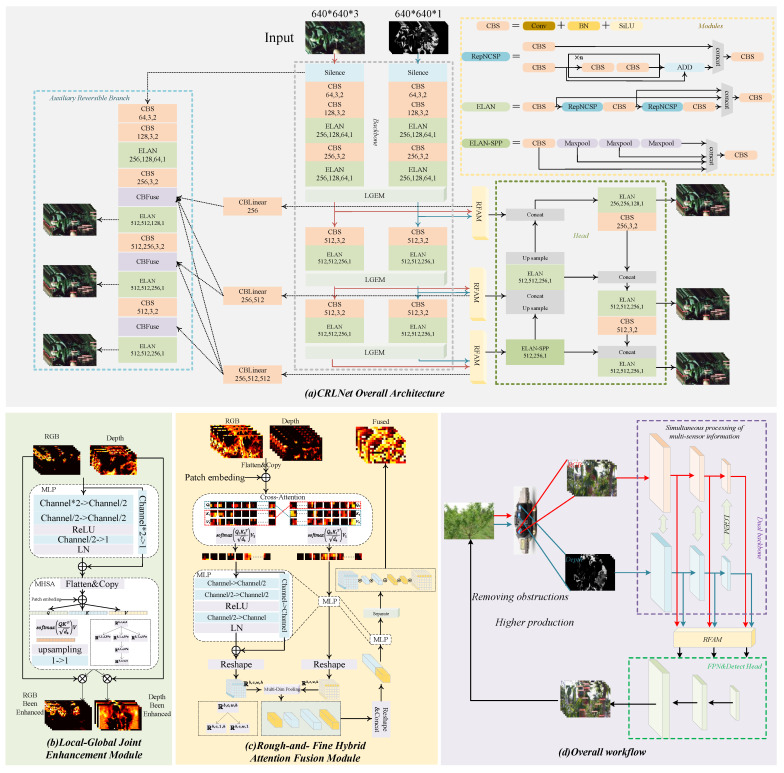
(**a**) The overall structure of CRLNet. The red line indicates the RGB information flow, the blue line indicates the depth information flow, the black line indicates the fusion information flow, the solid line indicates the main branch information flow, and the dashed line indicates the auxiliary branch information flow. (**b**) Overall structure of the LGEM. (**c**) Overall structure of the RFAM. (**d**) Workflow for using CRLNet in orchards.

The overall network architecture is presented in Figure 2a, where the numbers in the CBS indicate the number of channels of the outputs, the convolutional kernel size, and the number of module repetitions, respectively, from left to right. The four numbers in the ELAN module indicate from left to right the c2, c3, and c4 used for computation in the ELAN module, where the fourth number indicates the number of module repetitions. The numbers in the ELAN-SPP from left to right indicate the c2, c3, and the number of module repetitions used for computation, respectively. The arrays in the CBLinear module indicate the features that CBLinear processed into an array shape using linear transformation. Slience denotes the placeholder function, which is responsible for holding the input RGB information. Cblinear denotes the division of the features into equal parts using linear transformation, with the number of channels in each part determined by the numerical part under the module. CBFuse denotes the same scale at which the input features were first upsampled and then spliced on the channels. The yellow line in the figure indicates the visible information stream transmitted through the network, the blue line indicates the depth information, the black line indicates the fused multimodal information, and the dashed line indicates the information of the auxiliary stream.

The overall structure of the network consists of a dual-stream backbone and an auxiliary information flow, and the GELAN-based multimodal backbone is named dual GELAN. In the Dual-GELAN section, the number of channels of the input image is continuously expanded, and at different stages of the expansion, the features of different modalities are augmented using the LGEM. At the same time, the features of different stages are fused using the RFAM and then input into the shared detection head. Finally, the detection head uses the fused multimodal information to regress the categories of the objects and the bounding box to obtain the final detection result for a peach. In experiments, it was found that the best detection accuracy was achieved with RGB images under a single modal input; so, it was constructed as an auxiliary stream using RGB images to achieve further accuracy improvement using a smaller amount of computation. In the auxiliary stream, the features extracted from the dual-stream backbone are blended using RFAM and then fed into the CBLinear module; then, the blended features are separated into several features of different sizes using linear transformation. Finally, the separated features are fed into different stages of the auxiliary stream in order to enhance the information content of the auxiliary stream. The computational flow of CRLNet’s backbones is presented in Algorithm 1.

Figure 2d shows the overall workflow of the multimodal detection network proposed in this study. Firstly, the multimodal images are captured by the RGB and depth sensors; then, the multimodal images are input into the two-stream network equipped with RFAM and LGEM to perform the feature extraction. After that, the extracted fused features are input into the detection head to obtain the detection results, and finally, based on the results of the detection, the peach tree is pruned to ensure that the peach is not shaded.
**Algorithm 1** Calculation process of CRLNet**Require:** RGBimages and Depthimages **Ensure:** size(RGB)=size(Depth)
 1:**for** i=1 to 3 **do**▹ Feature extraction: 2:  $RGB,Depth\leftarrow backbone_{RGB}^{Stage_{i}},backbone_{Depth}^{Stage_{i}}$▹ LGEM: 3:  $Local\leftarrow concat\left(RGB,Depth\right)$ 4:  $E_{Local}\leftarrow LN\left(MLP(Local)\right)$ 5:  $q,k,v\leftarrow Flatten\left(E_{Local}\right),Flatten\left(E_{Local}\right),Flatten\left(E_{Local}\right)$ 6:  $Attention\leftarrow softmax\left(\frac{q\cdot k^{T}}{\sqrt{d_{k}}}\right)\cdot v$ 7:  $E_{Global}\leftarrow Conv_{1\to 1}\left(Upsampling\left(Attention\right)\right)$ 8:  $RGB,Depth\leftarrow RGB⊗E_{Global},Depth⊗E_{Global}$▹ RFAM: 9:  $q_{1},k_{1},v_{1}\leftarrow Flatten\left(RGB\right),Flatten\left(RGB\right),Flatten\left(RGB\right)$10:  $q_{2},k_{2},v_{2}\leftarrow Flatten\left(Depth\right),Flatten\left(Depth\right),Flatten\left(Depth\right)$11:  $Attention_{1}\leftarrow softmax\left(\frac{q_{1}\cdot k_{2}^{T}}{\sqrt{d_{k}}}\right)\cdot v_{2}$12:  $Attention_{2}\leftarrow softmax\left(\frac{q_{2}\cdot k_{1}^{T}}{\sqrt{d_{k}}}\right)\cdot v_{1}$13:  $RGB_{R},Depth_{R}\leftarrow Reshape\left(MLP\left(Attention_{1}\right)\right),Reshape\left(MLP\left(Attention_{2}\right)\right)$14:  $RGB_{w},RGB_{h}\leftarrow Maxpool_{w}\left(RGB_{R}\right),Maxpool_{h}\left(RGB_{R}\right)$15:  $Depth_{w},Depth_{h}\leftarrow Maxpool_{w}\left(Depth_{R}\right),Maxpool_{h}\left(Depth_{R}\right)$16:  $RD_{whwh}\leftarrow concat\left(RGB_{w},Depth_{w},Trans\left(RGB_{h}\right),Trans\left(Depth_{w}\right)\right)$17:  $RGB_{w},Depth_{w},Trans\left(RGB_{h}\right),Trans\left(Depth_{h}\right)\leftarrow split\left(MLP\left(RD_{whwh}\right)\right)$18:  $Fused_{i}\leftarrow RGB_{R}⊛RGB_{w}⊛RGB_{h}+Depth_{R}⊛Depth_{w}⊛Depth_{h}$19:**end for**


### 2.3. Local–Global Joint Enhancement Module (LGEM)

The overall structure of the LGEM is shown in Figure 2b, which consists of a local feature enhancement module in the first half and a global feature enhancement module in the second half. For the local feature enhancement module, it first fuses the features of different modalities:

(1) $$Local=concat\left(RGB,Depth\right),$$

where *RGB* denotes the features extracted from the *RGB* branch of the dual-stream network, and *Depth* denotes the features extracted from the depth branch of the dual-stream network. Next, the blended features are compressed using an *MLP* layer of a pure CNN:

(2) $$E_{Local}=MLP\left(Local\right),$$

where MLP(·) denotes the multilayer perceptron, and $E_{Local}$ is the weight used for local feature enhancement. It is worth noting that $E_{Local}\in R^{b,1,h,w}$, $Local\in R^{b,2c,h,w}$, and $RGB,Depth\in R^{b,c,h,w}$. In a concrete step, the inputs to the *MLP* layer are defined as *x*, the outputs are defined as *y*, and the specific calculation process of *MLP* is

(3) $$y=LN\left(Conv_{\frac{c}{2}\to 1}\left(ReLU\left(Conv_{\frac{c}{2}\to \frac{c}{2}}\left(Conv_{2c\to \frac{c}{2}}\left(x\right)\right)\right)\right)\right)⊕Conv_{2c\to 1}\left(x\right),$$

where the first term of the equation represents the channel of the feature compressed to 1 after a series of convolutional layers, LN(·) represents layer normalization, ReLU(·) represents the activation function, and $Conv_{p\to q}$ denotes a convolutional layer with input channel p, output channel q, convolutional kernel size 3, step size 1, and padding 1. The second term of the equation represents the residual, which avoids the potential loss of information that exists in the MLP during the compression of the channel, and ⊕ denotes the addition of corresponding positions in space.

The above steps use a purely convolutional structure to obtain $E_{Local}$, and due to the limitations of the convolution operation itself, $E_{Local}$ can only focus on localized detailed information. The advent of the Transformer [37] compensates for the lack of convolutional operations. However, the Transformer architecture has a high computation cost, which leads to a reduction in the speed of the network. Therefore, this study transformed the underlying Transformer architecture and proposed a global feature enhancement module as the second part of the LGEM. Specifically, the local weights are first transformed to compress them into vectors:

(4) $$q,k,v=Flatten\left(E_{Local}\right),Flatten\left(E_{Local}\right),Flatten\left(E_{Local}\right),$$

where Flatten(·) denotes replanning the shape of the feature. Specifically, the pre-planning dimension is $R^{b,c,h,w}$, and the post-planning dimension is $R^{b,1,c,h*w}$. Next, self-attention is computed on the planned vector:

(5) $$Attention=softmax\left(\frac{q\cdot k^{T}}{\sqrt{d_{k}}}\right)\cdot v.$$

Then, the features after the computation of self-attention are upsampled, and a miniature MLP layer is used to add further transformations to the results after the self-attention computation:

(6) $$E_{Global}=Conv_{1\to 1}\left(Upsample\left(Attention\right)\right),$$

where Upsample(·) is the inverse of Flatten. Finally, the enhanced features are obtained by augmenting the RGB and depth features using the global weight map:

(7) $$RGB_{E},Depth_{E}=RGB⊗E_{Global},Depth⊗E_{Global}.$$

The heat maps of the feature maps before enhancement and after enhancement are presented in Figure 2b. It can be seen that, after the LGEM processing, the information of different modalities becomes more delicate. The RGB part of the network focuses more attention on the peach, and the depth map part focuses attention on the part where the depth information is concentrated.

### 2.4. Rough–Fine Hybrid Attention Fusion Module (RFAM)

Using simple summation or splicing on channels does not mix the features of different modalities well. Numerous studies have shown that designing a reasonable architecture to fuse features of different modalities can better exploit the multimodal information [36,38]. In this study, the RFAM was designed to fuse features of different modalities based on the framework of coarse fusion first and fine fusion later. Figure 2c illustrates the overall architecture of the RFAM. First, the input features are spread as vectors:

(8) $$q_{1},k_{1},v_{1}=Flatten\left(RGB\right),Flatten\left(RGB\right),Flatten\left(RGB\right)$$

(9) $$q_{2},k_{2},v_{2}=Flatten\left(Depth\right),Flatten\left(Depth\right),Flatten\left(Depth\right).$$

Next, the cross-attention of the different modalities is calculated:

(10) $$Attention_{1}=softmax\left(\frac{q_{1}\cdot k_{2}^{T}}{\sqrt{d_{k}}}\right)\cdot v_{2}$$

(11) $$Attention_{2}=softmax\left(\frac{q_{2}\cdot k_{1}^{T}}{\sqrt{d_{k}}}\right)\cdot v_{1}.$$

Then, two mutually independent MLPs are used to add further transformations to the self-attention computed features, followed by reshaping the transformed features into the same shape as the input to obtain the coarsely fused visible and depth features:

(12) $$RGB_{R},Depth_{R}=Reshape\left(MLP\left(Attention_{1}\right)\right),Reshape\left(MLP\left(Attention_{2}\right)\right),$$

where $RGB_{R}$ and $Depth_{R}$ denote the RGB and depth features after coarse fusion, respectively. Reshape(·) is the inverse of Flatten(·). Specifically, defining the input features of the MLP layer here as *x* and the output features as *y*, it can be concluded that

(13) $$y=LN\left(Conv_{\frac{c}{2}\to c}\left(ReLU\left(Conv_{\frac{c}{2}\to \frac{c}{2}}\left(Conv_{c\to \frac{c}{2}}\left(x\right)\right)\right)\right)\right)⊕Conv_{c\to c}\left(x\right).$$

Next, the fine-tuning of the coarsely fused features continues by first performing maximum pooling on the *H* dimension and the *W* dimension for $RGB_{R}$ and $Depth_{R}$, respectively, such that the information in different dimensions is compressed into vectors, and the specific process can be formulated as

(14) $$RGB_{w},RGB_{h}=Maxpool_{w}\left(RGB_{R}\right),Maxpool_{h}\left(RGB_{R}\right)$$

(15) $$Depth_{w},Depth_{h}=Maxpool_{w}\left(Depth_{R}\right),Maxpool_{h}\left(Depth_{R}\right),$$

where $RGB_{w}$ and $RGB_{h}$ denote the RGB feature vectors compressed in the *w* and *h* dimensions. $Depth_{w}$ and $Depth_{h}$ denote the same operation taken for depth features, where $RGB_{w},Depth_{w}\in R^{b,c,w,1},$ and $RGB_{h},Depth_{h}\in R^{b,c,1,h}$. Next, the last two dimensions of $RGB_{h}$ and $Depth_{h}$ are exchanged and then spliced with $RGB_{w}$ and $Depth_{w}$ to obtain the blended feature vector:

(16) $$RD_{whwh}=concat\left(RGB_{w},Depth_{w},Trans\left(RGB_{h}\right),Trans\left(Depth_{w}\right)\right),$$

where $RD_{whwh}$ denotes the feature vector after blending, and $Trans\left(\cdot \right)$ denotes swapping the last two dimensions of the matrix. Immediately after that, an MLP layer identical to the rough fusion is used to add learnable parameters to the blended feature vectors; then, the processed blended feature vectors are separated, and the processes of separation and merging are reversible:

(17) $$RGB_{w},Depth_{w},Trans\left(RGB_{h}\right),Trans\left(Depth_{h}\right)=splite\left(MLP\left(RD_{whwh}\right)\right).$$

In this way, the features of different modalities are blended at different granularities; finally, the learned parameters are used for the final step of fine fusion:

(18) $$Fused=RGB_{R}⊛RGB_{w}⊛RGB_{h}+Depth_{R}⊛Depth_{w}⊛Depth_{h},$$

where ⊛ denotes the matrix multiplication. The RFAM first uses cross attention to coarsely fuse features from different modalities, followed by fine-tuning the coarsely fused features using different dimensions of attention. The usefulness of the RFAM is demonstrated in the experiments; moreover, the RFAM module can be generalized to any multimodal fusion problem.

## 3. Experimental Results and Analysis

This section begins with a description of the experimental setting in Section 3.1; the metrics used to quantitatively compare the different methods are presented in Section 3.2; the model results are presented and analyzed in Section 3.3; the ablation experiments are described in Section 3.3.6, confirming the validity of the RFAM and the LGEM; the method of the present work is compared with the state-of-the-art methods in Section 3.3.7; and generalization experiments are described in Section 3.3.8.

### 3.1. Experimental Environment

The experimental environment is shown in Table 1, and the training data were processed using mosaic enhancement to ensure that the peaches were uniformly distributed anywhere in the image. The mosaic enhancement was turned off in the last 30 training rounds, which sped up the convergence of the model. The mosaic enhancement was performed simultaneously on both the RGB and depth images to ensure that the enhanced image remained strictly aligned.

### 3.2. Evaluation Metrics

To make a quantitative comparison of CRLNet, *P*, *R*, and *mAP* were used as the evaluation indices, where *P* denotes the accuracy rate, which is calculated by the formula

(19) $$Precision=\frac{T_{P}}{T_{P}+F_{P}},$$

where $T_{P}$ denotes that the model successfully detects positive samples as positive samples, and $F_{P}$ denotes that the model predicts negative samples as positive samples. R denotes the recall rate, which is calculated as follows:

(20) $$Recall=\frac{T_{P}}{T_{P}+F_{N}},$$

where $F_{N}$ is the probability that the model detects a positive sample as a negative sample. The mAP is the *AP* of each category divided by the total number of categories, and the specific formula for *AP* is

(21) $$AP=\frac{T_{P}+T_{N}}{T_{P}+T_{N}+F_{P}+F_{N}},$$

where $T_{N}$ denotes the probability of correctly predicting a negative sample as a negative sample, where the criterion for judging correct and incorrect is the IOU. When the IOU of the prediction box and the GT box is greater than a set threshold, it is judged to be correctly predicted; otherwise, it is judged to be incorrect. Based on different IOU thresholds, we can obtain the mAP, where $mAP_{50:95}$ represents the average result of the mAP calculated with a step size of five, ranging from a threshold of 50 to 95.

In addition, to quantitatively compare the size of the models, we count the total number of parameters required to train the models, Params, where a smaller number of parameters indicates a lighter model. To visualize the speed of the network, we also count the time taken by different models to process one or a pair of input images, denoted as Speed, where Params consists of the number of parameters in the Conv and FC layers, respectively:

(22) $$Params_{Conv}=\left(K\times in+1\right)\times out,$$

where *K* is the convolution kernel size, in is the number of feature channels in the input convolution layer, and out is the number of feature channels in the output convolution layer. Similarly, the number of parameters in the FC layer is calculated as

(23) $$Params_{FC}=\left(in+1\right)\times out,$$

where in is the length of the feature vector of the input FC layer, and out is the length of the feature vector of the output FC layer. The total Params are calculated as

(24) $$Params=Params_{Conv}+Params_{FC}.$$

To make the measurement of the network speed more accurate, selected *N* samples are counted as the total time *T* to process the samples, obtaining the time needed to process each sample speed by calculating

(25) $$speed=\frac{T}{N},$$

where *T* is in milliseconds.

### 3.3. Results and Analyses

#### 3.3.1. Data Distribution

**Figure 3:**
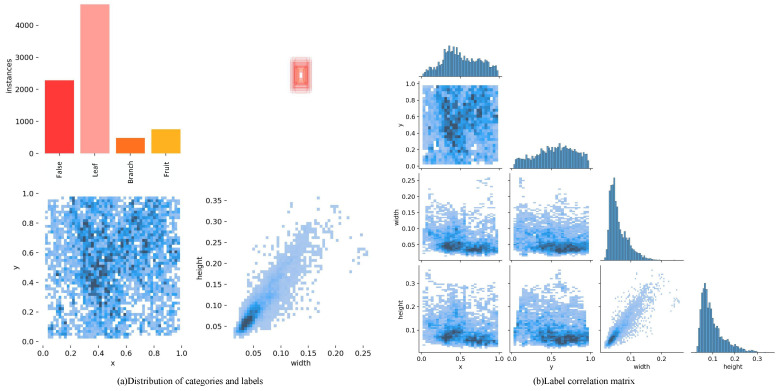
The distribution of data in the training dataset. (**a**) The number and size of different categories of peaches in the dataset. (**b**) Modeling of the correlation between tags using the target detection algorithm during training, where the darker the color, the higher the correlation.

The dataset was divided into three distinct subsets, designated as training, validation, and testing sets, in a ratio of 8:1:1. Figure 3 illustrates the data distribution of the dataset. In Figure 3a, the top-left plot indicates the number of labels for each category, where “False” indicates unshaded peaches, “Leaf” indicates peaches shaded by leaves, “Branch” indicates peaches shaded by branches, and “Fruit” indicates peaches shaded by fruits; the top-right corner shows the shapes of all the target frames. The ground truth in the YOLO format consists of x, y, w, and h, where x and y denote the coordinates of the box, and w and h denote the dimensions of the box. The horizontal coordinates of the bottom-left subplot in Figure 3a indicate the values of x, and the vertical coordinates indicate the values of y. The data in the plot indicate that the peaches of the dataset were concentrated in the central region of the image. The lower-right subplot in Figure 3a indicates the value of w in horizontal coordinates and the value of y in vertical coordinates, and the data in the figure indicate that the target frame of peaches in the dataset was small. Figure 3b shows the degree of association between x, y, w, and h in detail. In each of these subplots, the horizontal and vertical coordinates indicate the specific value of each variable. Darker cells indicate that the model learned the correlation between the two labels well. Light-colored cells, on the other hand, indicate a weaker correlation.

The data presented in Figure 3 indicate that a significant proportion of the peaches in the dataset were shaded by leaves, while only a small number were shaded by branches and trunks. This uneven distribution of samples poses a challenge in achieving a higher detection accuracy. In the analysis of the detection frame, it can be observed that the peaches that require detection in this study are concentrated in the central region of the image, and they possess a smaller target frame. It is also noteworthy that the young peaches are relatively small, and the different occlusions tend to have similar morphological characteristics. Furthermore, RGB-based detection algorithms tend to confuse different occlusions, and the introduction of depth information can assist the model in more accurate classification.

#### 3.3.2. Modal Combination Analysis

The research in this study combined RGB and depth. In fact, infrared data also exist in the data used in the datasets, and this study carried out experiments on the combination of different modes of information using these three types. The experimental data are presented in Table 2, where the first three rows are unimodal information. The data show that in the unimodal information combination, the RGB and RGB achieved a $mAP_{50}$ of 95.73% because the RGB information was sufficient for most of the scenes. Depth and IR only achieved $mAP_{50}$ of 85.24% because these two types of information were more suitable for complementing and extending the RGB information rather than being input as the main information into the network. The multimodal combination of information again confirmed the suitability of the depth and IR information as complementary information. In the multimodal comparison, the combination of RGB and depth and the combination of RGB and IR achieved a certain degree of improvement in accuracy; yet, the value of the improvement did not fully confirm that the combination of RGB and depth had a great advantage. However, when the module in CRLNet was embedded in a network, compared with the combination of RGB and IR, the $mAP_{50}$ and $mAP_{50:95}$ increased by 1.93% and 8.3%, respectively, in the combination of RGB and depth. This situation arises because infrared images can detect information about objects that emit heat radiation, and it is clear that the heat radiation from peaches was almost negligible. As crops emit limited heat, the combination of RGB and depth is the most applicable in the field of agriculture. In areas such as pedestrian detection, the combination of RGB and IR can provide a large boost.

**Figure 4:**
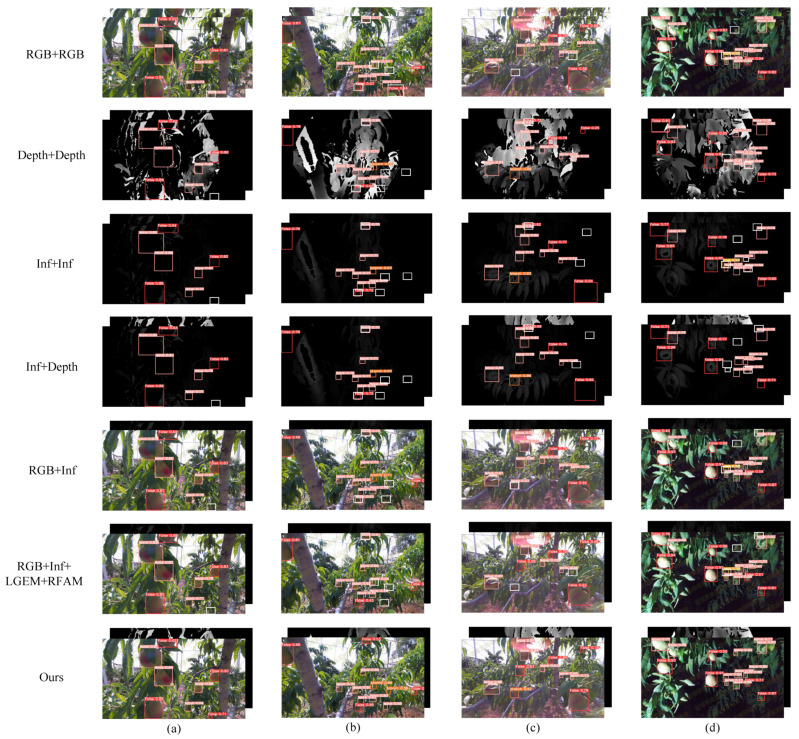
Comparison of the detection results for different combinations of modes. (**a**) normal brightness; (**b**) dense peach; (**c**) glare interference; (**d**) dark light.

A comparison of the detection results for different modal combinations is provided in Figure 4. Wrongly detected and missed targets are marked with white boxes in the figure. Coinciding with the data in Table 2, under the combination of Inf + Inf and Depth + Depth, the $mAP_{50}$ is only 85.91% and 83.43%, respectively, and the data in the figure also show that the infrared image provided almost no information. The RGB images have almost no missed or false detections in scenes with normal brightness, but when the environment became harsh, the RGB image started to fail. The combination of RGB and depth achieved stable and high-accuracy detection, and the accuracy of detection was further improved after embedding the module in this study.

**Figure 5:**
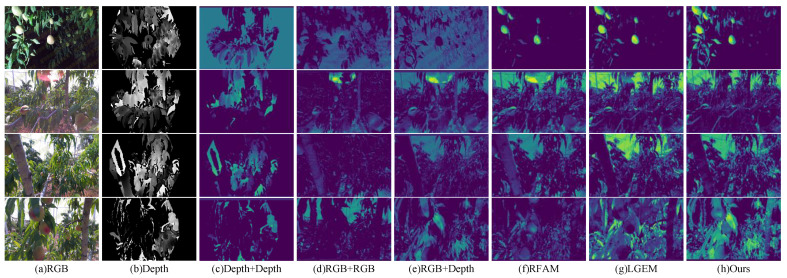
Extracted feature maps with different input information, where warmer colored regions indicate that the network is focusing more attention on that region and the opposite is true for cooler colored regions.

To explore the feature expressiveness of the network under different types of input information, Figure 5 shows the feature heat maps of several different classes of input sources after feature extraction from the backbone network. The figure shows that for a single modal input, the network could not focus on the peach region. The combination of RGB + Depth moved the network’s attention to the peach region, and the attention was more focused after the embedding of the enhancement module. The embedding of the fusion module allowed for more information to be introduced into the depth map, and the number of peaches observed became larger. The method used in this study allowed for full attention to be focused on the peach region and filtered out the distracting background information.

Existing consumer-grade sensors can capture RGB, depth, IR, and other images simultaneously. To explore the effect of more modalities on the peach detection accuracy, further modalities were combined. Specifically, CRLNet was extended to three streams to process the information from three models simultaneously. To allow for the module in this study to accommodate information from more modalities, the RFAM and LGEM were adapted to define three different modalities *x*, *y*, and *z*. Then, the fused information *f* was defined as

(26) $$f=RFAM\left(RFAM\left(x,y\right),RFAM\left(x,z\right)\right)$$

For the LGEM module, we adjusted Equation (1), which was defined as

(27) $$Local=concat\left(x,y,z\right).$$

Correspondingly, the input channels of the MLP layer changed. The specific metrics of the three-stream network are presented in Table 3, and the data show that the three-stream network did not improve the accuracy; in contrast, the final $mAP_{50}$ is 1.3% lower than the RGB + Depth combination. This is because in agriculture, images taken by IR cameras do not reflect the information well, and extracting features using different feature extraction networks and fusing them using the same fusion module will result in less informative IR features interfering with the RGB and depth features, causing a decrease in accuracy. One possible way to utilize the three streams of information would be to design an appropriate fusion module to filter the IR information; another possible way would be to simultaneously input the information from the three modalities into a single stream detection network using input-level fusion. The best results can be achieved by selecting the appropriate modal information and constructing the proper network structure according to the specific application scenario.

#### 3.3.3. Efficiency of the RFAM

The RFAM consists of a coarse fusion phase based on cross attention and a fine-tuning phase based on multidimensional mixed attention. To illustrate the effectiveness of the coarse–fine fusion strategy in the RFAM, the results of the ablation experiments conducted on the RFAM are presented in Table 4, where NO denotes a multimodal network that does not use any module, RAM denotes a multimodal network that uses only the coarse fusion module, FAM is a network that uses only the fine fusion module, and RFAM is a network that is embedded with the fusion module of this study. The data show that the use of either fusion method alone leads to degradation in the network performance due to the presence of a large amount of interfering information in the peach background. If the multimodal information is fused without adopting an appropriate fusion strategy, the fused features contain too much interfering information, thus leading to degradation in the network accuracy. Furthermore, this phenomenon also shows that simple attention-based fusion rules cannot achieve excellent results.

In the fine fusion stage of the RFAM, a fusion method based on multidimensional attention was proposed in order to exclude the positive effect of the attention mechanism on the accuracy of the network and to confirm the effectiveness of the fine fusion stage of this study. The FAM part of the network used in this study was replaced with several different attention mechanisms in turn, and a comparison of their effectiveness is presented in Table 5, where NO denotes a multimodal network without embedded modules. When using ResCBAM [39] as a fine fusion strategy, $mAP_{50:95}$ significantly decreased by 6.89% due to the fact that too much attention computation corrupts the coarsely fused features. When embedding SE [40] and ECA [41], which have relatively simple structures, compared with single stream networks, $mAP_{50:95}$ was increased by 4.47% and 3.26%, respectively; however, they could not achieve the same effect as the FAM proposed in this study, which confirms that the FAM part of the CRLNet is reasonable and effective in the peach detection task.

#### 3.3.4. Performance of CRLNet at Different Resolutions

**Figure 6:**
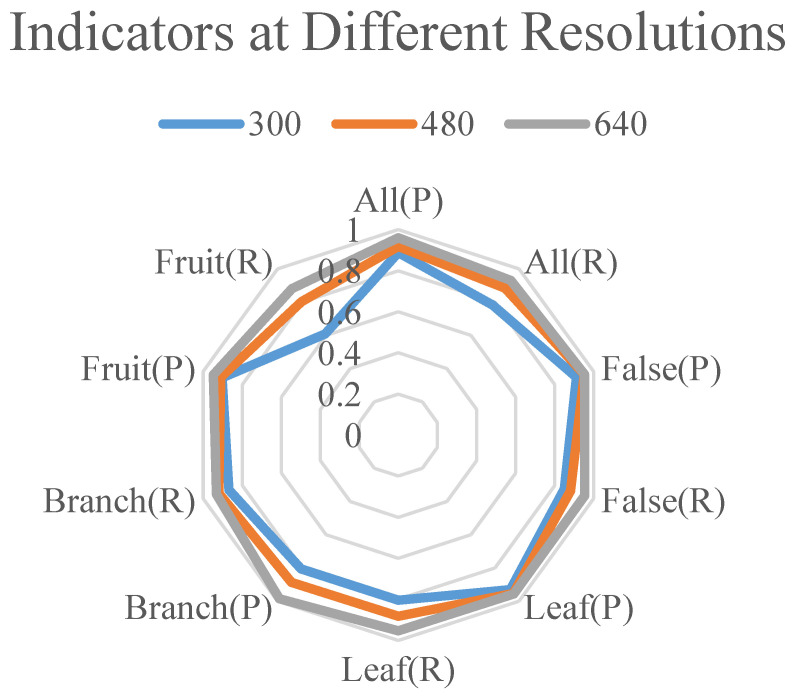
P and R for different classes of CRLNet at different resolutions.

The resolution of the detection model has a strong impact on the accuracy of the model. The detection accuracy under 320 × 320, 480 × 480, and 640 × 640 input image sizes was tested, respectively. As shown in Table 6, the mAP increased gradually as the resolution increased. The model reached equilibrium when the resolution was 640 × 640, as shown in Figure 6. There was no need to use higher-resolution images in this study because too high a resolution leads to an increase in the computational complexity and a decrease in the percentage of the model’s receptive field in the image, which can affect the model’s detection ability.

#### 3.3.5. Analysis of Detectors of Different Sizes

**Figure 7:**
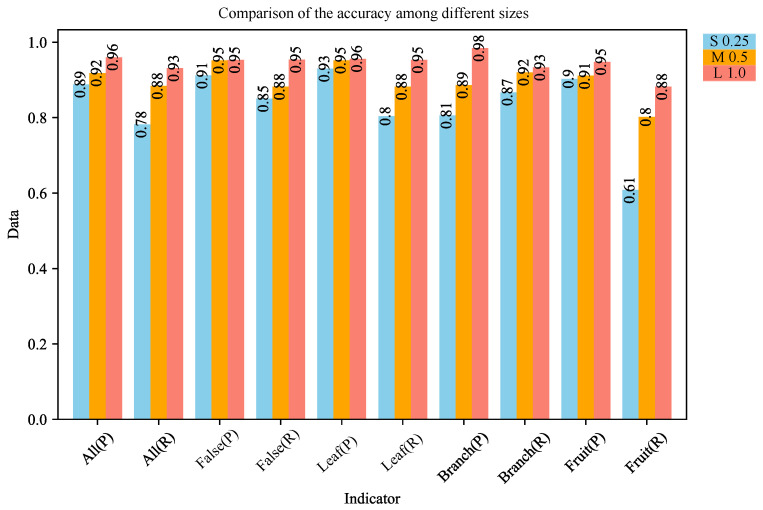
Comparison of P and R metrics for different sizes of detectors.

The existing detectors are published in different sizes to cope with different deployment scenarios. In this study, two lightweight versions were provided, s and m, in addition to the basic model by scaling the channels and width of the network at each layer. A comparison of the metrics of the different versions is shown in Table 7. The data show that at different scales, the model in this study achieved at least 5% and 5.97%$mAP_{50:95}$ improvement compared to the single stream model in these two cases, respectively. More specifically, further metric comparisons for different sizes of detectors are provided in Figure 7. In different scenarios, suitable detectors can be chosen for different tasks. For example, for a stationary peach detector with a stable power supply, the l version can be used; for a small camera with insufficient voltage, the lightweight m version can be used; for a UAV orchard inspection, the lightest s version can be used.

#### 3.3.6. Ablation Experiment

**Figure 8:**
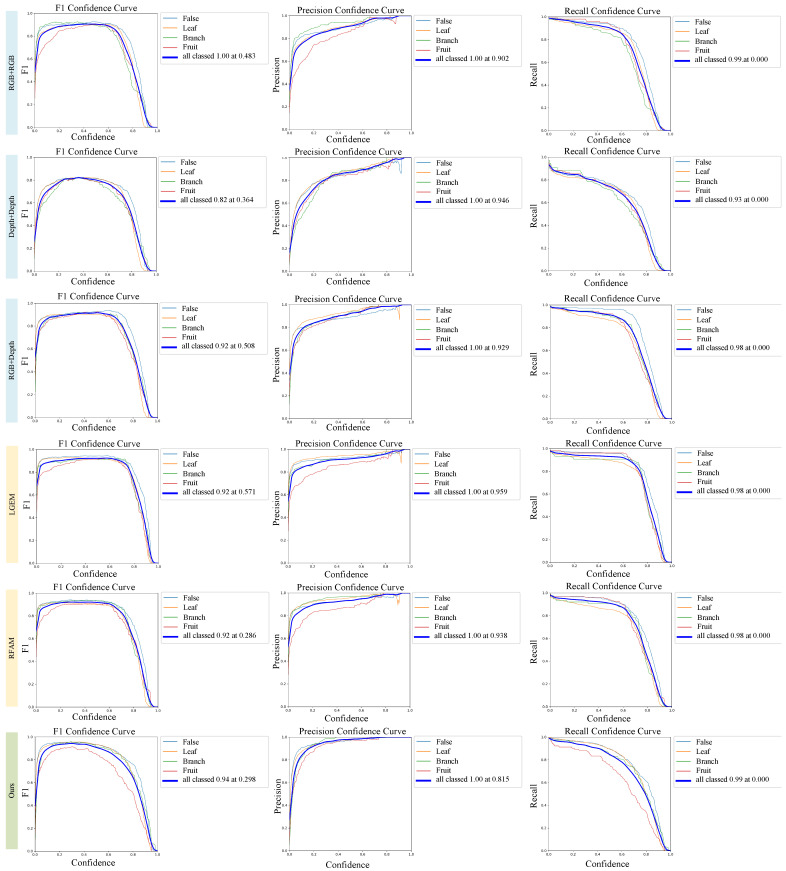
*P*, *R*, and *F1* transformation curves for different methods at different confidence levels. where the horizontal coordinate of each subplot indicates the specific value of the confidence level and the vertical coordinate indicates the specific value of the indicator. Different colors indicate different categories, and the data in the legend indicate the best indicator at the best confidence level.

To confirm that the model in this study is the optimal solution for engineering in terms of the data selection, module design, and architecture design, ablation experiments were conducted on CRLNet, and the experimental design and related data are shown in Table 8. In the unimodal mode, the RGB image provided the most abundant feature information. However, the multimodal model had a significant advantage over the unimodal model. Simple channel splicing feature fusion methods may introduce low-quality depth information that interferes with the spectra, which, in turn, leads to a reduction in the detection accuracy. The LGEM was designed to improve the quality of the RGB and depth information simultaneously. In addition, an RFAM based on the transformer architecture was designed, and it effectively integrates the advantageous information from both modalities. These results highlight the importance of feature-level fusion-based approaches in multimodal detection tasks in agricultural scenes. Through embedding the LGEM and RFAM into the dual-stream detection model designed based on YOLOv9, the overall performance of CRLNet significantly improved, especially in the $mAP_{50:95}$ metric, which improved by 6.93%. In addition, to complement and corroborate the data in Table 8, we also present the variation curves of the P, R, and F1 values of the different methods at different confidence levels in Figure 8, where the blue curves represent the average accuracy of all categories, and the curves of different colors represent the specific accuracy of different categories. In the legend section, the best metrics achieved by the different methods at the best confidence level are shown. The data show that CRLNet can achieve the best accuracy under lower confidence thresholds, proving that ours method can achieve accurate localization and classification of different classes of peaches.

**Figure 9:**
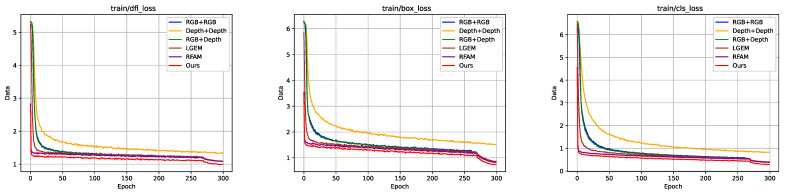
Plot of the training loss variation for different data combinations and different module combinations.

Typically, networks with a larger number of parameters tend to have convergence problems, and different input data may impact the convergence of the model. To verify that the modules as well as the data used in this study did not have a negative impact on the convergence of the model, we show the graph of the variation in the training loss for different data combinations and different module combinations in Figure 9. The data in the figure show that comparing the different input sources, the combination of RGB + Depth achieved the lowest loss value, and the combination of Depth + Depth achieved the highest loss value, which also matches the data from the accuracy analysis. In the comparison of embedding different modules, the model in this study achieved the lowest loss and the fastest convergence speed, which confirms that it has a positive impact on the convergence speed and accuracy of the network. The loss profile shows a rapid drop in the last 30 training rounds; this was due to the mosaic enhancement technique being turned off.

#### 3.3.7. Comparative Experiments with State-of-the-Art Algorithms

CRLNet was tested on the multimodal peach dataset and compared with other state-of-the-art target detection algorithms. The single-stream target detection frameworks include YOLOv3, YOLOv5, YOLOv6, YOLOv8, YOLOv9, and RT-DETR, and the dual-stream detection frameworks compared include CMASF [34], AFM [32], SCA [42], and MIA [43]. All of these single-stream comparison algorithms used the standard version, and for a fair comparison, the state-of-the-art detection model YOLOv9 was used as the baseline model for these dual-stream comparison algorithms with the embedded modules, as proposed in the original paper. As can be seen from Table 9, the highest accuracy in the comparison of unimodal networks was achieved using RGB images, which in most cases reflects the scene information well. Compared to YOLOv9, which has the best accuracy in unimodal networks, CRLNet was improved by 1.02% and 6.93% on $mAP_{50}$ and $mAP_{50:95}$, respectively. In addition, compared to the SCA, the detection network with the second best accuracy in multimodality, CRLNet, achieved a 1.65% and 5.33% improvement in $mAP_{50}$ and $mAP_{50:95}$, respectively. This indicates that CRLNet detected peaches more accurately and was more robust to different IOU thresholds. CRLNet also achieved a better balance between detection accuracy and computational complexity without introducing too many additional parameters, which can still satisfy real-time detection.

**Figure 10:**
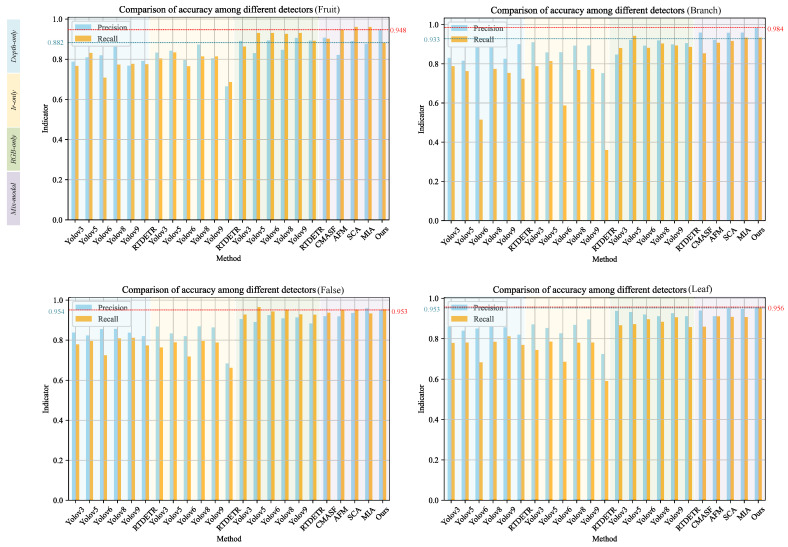
Comparison of the accuracy (P) and recall (R) of each detection model for different occlusion categories of peaches. The four color regions from left to right in each sub-figure correspond to depth-only, IR-only, RGB-only, and mixed.

Similarly, the accuracy and recall of different models for detecting different categories of peaches were compared and analyzed. Depending on the type of input data, the detection models can be classified into four broad categories, namely, those using only RGB images, those using only infrared images, and those using only depth images versus those using mixed data. As can be seen in Figure 10, the red and blue dashed lines represent the values of P and R obtained by CRLNet. CRLNet achieved the highest accuracy among all models for different occlusion cases of peaches, with an average accuracy of 96.01%, and CRLNet also achieved a significant improvement over other two-stream networks. The experimental results show that CRLNet achieved impressive performance in both precision and recall. This excellent performance is attributed to the design of our fusion method, which effectively combines depth information to complement RGB image features, enabling it to excel at challenging problems such as complex backgrounds, occlusions, and targets of different scales.

**Figure 11:**
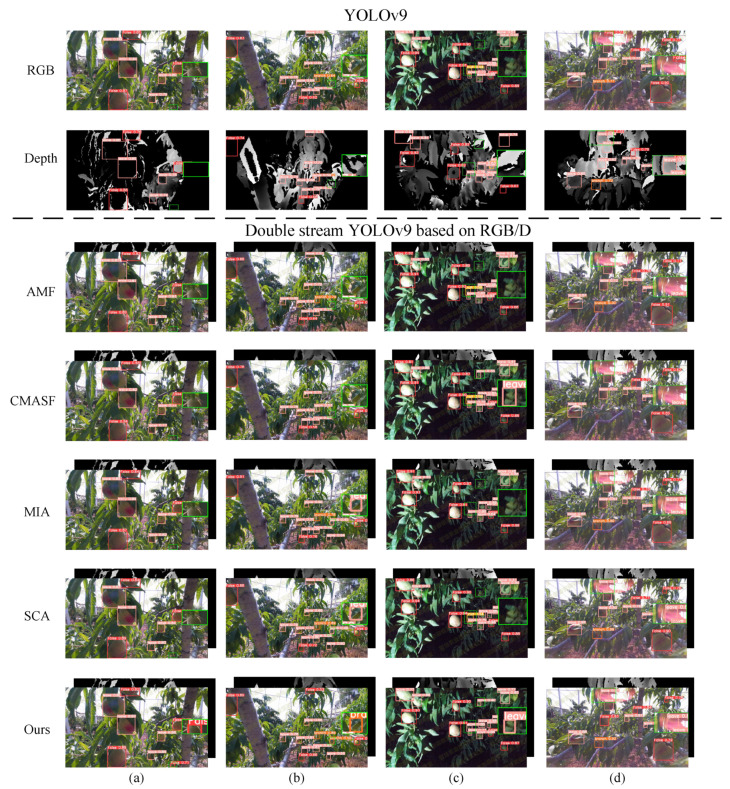
Visualization of the state-of-the-art single- and dual-stream detection algorithms on the peach dataset: (**a**) normal brightness; (**b**) dense peach; (**c**) dark light; (**d**) glare interference.

In addition, to visually demonstrate the effectiveness of the models in this study, the visual detection results of the state-of-the-art single-stream detector YOLOv9 compared to other state-of-the-art dual-stream detectors are shown in Figure 11. typical scenes under three different lighting conditions were selected: sunny days, glare, and artificial lighting at night. The missed and false detections of the single-stream YOLOv9 and other dual-stream algorithms relative to CRLNet are marked with green boxes and magnified to the right of the image. For a fair comparison, all the dual-stream algorithms compared in the experiments were implemented by embedding the modules of the original paper into the extended YOLOv9. As can be seen in Figure 11a, all the algorithms achieved good detection results under normal lighting on a sunny day, and the other algorithms (except for our method), only missed a small peach at one location at the bottom of the image. Figure 11b shows a sunny day scene with some peaches blended into the background, where CRLNet successfully detects the peaches, unlike the other methods. Figure 11c shows a flare scene, where the red flare caused by the lens directly facing the sunlight interferes with the color characteristics of the peaches, and both the original YOLOv9 and some of the dual-stream detection algorithms failed to detect them. Figure 11d shows the artificial lighting scene at night, where all the methods except ours had different degrees of misdetection and omission. This shows that CRLNet not only has good detection performance under normal lighting conditions but also has a stronger feature capture and anti-interference ability than other algorithms under extreme lighting conditions.

#### 3.3.8. Generalization Experiment

Ripe peaches often need to be bagged during the picking period to prevent birds from nibbling them, and it is equally important for picking robots to achieve the accurate localization of bagged peaches. To verify the effectiveness of the CRLNet proposed in this study in detecting bagged peaches, the bagged peach dataset (https://download.scidb.cn/download?fileId=62cc068edd6f884c9c9b9c7d (accessed on 9 July 2022)). was used for re-training and testing. The bagged peach dataset has only one category and has more image pairs. The training process used the same data partitioning and training parameters as the young peach detection, and to speed up the experiments, the s version of the detector was used for training and validation. The performance indicators are shown in Table 10. When embedding CMASF, MIA, and SCA, $mAP_{50:95}$ actually decreased by 3.17%, 2.67%, and 2.8%, respectively, confirming that these methods do not have strong generalization capabilities. CMASF uses an attention-based approach to fusion, and the attention-based approach is generic and therefore not overly affected when the data distribution changes. The module in this study also used some attention mechanism in its design, and due to the granularity refinement of the fusion rules used in this study, CRLNet still showed state-of-the-art performance in the detection of bagged peaches, once again demonstrating its robustness and generalizability.

## 4. Discussion

Accurate positioning of peaches is crucial for automated orchard management. A large number of studies have demonstrated that accurate positioning of peaches can be achieved using deep learning-based approaches, among which YOLO-based methods have been most widely used [1,7,8,9]. With further research, a large number of studies have shown that higher accuracy can be achieved by using multimodal data for peach detection [16]. These methods that use multimodal data usually select a suitable single-stream detector for expansion, followed by expanding the backbone of the single-stream detector into a dual-stream to adapt to the multimodal inputs, and then fusing the multimodal features using summation or channel splicing immediately afterwards, and finally using a common detector head to regress the fused features to obtain the final detection results. These studies have neglected the negative impact of unbalanced multimodal information on the model during the fusion process. In addition, uncontrolled light in orchards has not received sufficient attention. To this end, this study proposes CRLNet, selects the advanced unimodal detector YOLOv9 for expansion, and designs RFAM, a multimodal feature fusion module with progressive granularity, along with LGEM, which mitigates the interference of uncontrolled light. These enhancements achieve the best results across various peach detection tasks.

In this study, the well-known single-stream detectors YOLOv3 [22], YOLOv5 [16], YOLOv6 [44] YOLOv8 [23], YOLOv9 [24], and RT-DETR [45] were selected for metrics comparisons with the dual-stream detectors CMASF [34], AFM [32], SCA [42], and MIA [43]. The data are shown in Table 9. In the comparison using different modalities, the best detection accuracy is achieved using RGB images alone, which is due to the fact that RGB images can present enough scene information in most scenes. In the single stream detector comparison, owing to the powerful feature extraction backbone GELAN, YOLOv9 achieves the best detection accuracy with an $mAP_{50}$ of 96.11%. In the comparison of dual-stream detectors, MIA fusion using mixed attention achieved the best accuracy with an $mAP_{50}$ of 95.67%; however, it was not as high as using YOLOv9 alone because of the large number of uncontrolled light interferences in the dataset taken for this study, which caused the conventional fusion module to fail. Owing to the strong effects of LGEM and RFAM, CRLNet achieved an $mAP_{50}$ of 97.13%, a 1.02% improvement compared to the best single-stream detector. On the more stringent $mAP_{50:95}$ comparison, the CRLNet accuracy reached 69.44%, which is a 6.93% improvement compared to the best single-stream detector, proving the effectiveness of the method in this study on the task of accurate peach localization.

Real orchard scenes are often affected by uncontrolled lighting, and existing research has focused on the characteristics of the peaches themselves, ignoring the effects of the scene on the detector. When harsh sunlight interferes in the scene, some regions in the RGB image cannot be recognized in the image with the naked eye, let alone using the detector for automatic detection. Therefore, we design LGEM based on joint local-global enhancement, which uses a lightweight CNN and a preliminary mixture of multimodal features for initial local modeling of the scene, followed by the introduction of a Transformer layer with global sensing capability for overall structure-selective enhancement, so as to repair the contaminated information in RGB. Similar conclusions have been reached in related studies [38].

Widely differing multimodal features are often not well fused by simple summation or on-channel splicing [46]. Existing work has recognized this and designed a number of attention-based fusion rules for blending multimodal information [32,34,42,43]. However, it is shown in the experimental section that these attention-based approaches did not perform satisfactorily in the peach detection task effect. This is due to the large overall target of peaches and the long distance between different peaches, the spacing region between two peaches does not have any information in the Depth image, resulting in a large number of voids in the Depth image. Furthermore, contaminated regions with insufficient information are present in the RGB image, which is affected by uncontrolled lighting. Simply using an attention mechanism to model multimodal information that is unbalanced, highly varied, and with significant missing information does not result in reasonable fusion weights. For example, CMASF [34] performs well in the intensive tea leaf detection task due to the fact that there is not a lot of null information in the Depth image of tea leaves. In this study, through the RFAM module, cross-modal feature aggregation is first performed for both RGB information and Depth information using a Transformer, which fills in the missing regions in both modalities at a semantic level. This is immediately followed by fine-tuning of the initially aggregated features using fusion rules based on the attention mechanism. In the analysis of existing studies, it was found that using one type of attention alone does not fuse features from different modalities as well as possible. For example, SCA [42], which uses both spatial and channel attention, achieves better detection accuracy than CMASF [34], which uses spatial attention alone. This is due to the fact that one type of attention alone cannot perform comprehensive feature screening. In addition, weight learning using a mixture of features is superior to weight learning using separate features of either modality. For example, MIA [43] achieves better results than AFM [32], which is due to the fact that weight learning using features of any one modality alone leads to an unbalanced bias of the fused features towards one modality, resulting in the advantage of multimodal information not being fully exploited. Therefore, in this study, we designed a fine fusion module based on coordinate attention mechanism, which firstly performs preliminary feature extraction for features of different modalities, followed by aggregation of features of different dimensions, and finally obtains the fusion weights through the learning of the aggregated features, which achieves fine-tuning of the coarse fusion features, thus achieving better detection accuracy. Similar studies have achieved similar results [35].

LGEM fixes light-polluted RGB features by combining global structural information and local detail information, which makes up for the shortcomings of existing studies in dealing with uncontrolled light problems and significantly improves the detection accuracy of CRLNet. The data in Table 8 show that the $mAP_{50:95}$ increased by 1.5% after embedding LGEM. To further improve the detection accuracy, this study summarizes the features of existing studies and proposes a granularity progressive fusion module RFAM with the powerful feature aggregation capability of the Transformer. Unlike existing attention-based fusion modules, RFAM first uses a Transformer to perform the initial spatial aggregation, followed by the fusion of the features using coordinate attention. Fine-tuning the data in Table 8 shows that $mAP_{50:95}$ increased by 1.52% after embedding RFAM. When the two modules proposed in this study are embedded simultaneously, $mAP_{50:95}$ increases by 6.93%, indicating that the two modules are not only effective, but also promote each other.

CRLNet achieved excellent results on the peach detection task. In future work, the algorithm can be combined with automated mechanical equipment to achieve automated pruning and fruit collection in the orchard. Specifically, the detection method proposed in this study can be used to accurately locate peaches, followed by accurate automated harvesting and pruning using a robotic arm. In the process of positioning, images are collected by configuring an aligned RGB camera and Depth camera on the robotic arm, followed by inputting the collected images into CRLNet to obtain the detection results, and finally manipulating the robotic arm to move by using the detection results with the control algorithm. In addition, the method in this paper can also be used for yield prediction in production. There are many peach trees in the orchard, and different peach trees do not have the same yield, so predicting the parameters before fruit ripening and assigning the right amount of manual or automated mechanical equipment to each peach tree can improve the operational efficiency of the orchard. Specifically, an aligned multimodal camera can be set up next to a fruit tree to automatically collect images of a pair of peach trees every day, followed by uploading the collected images to the server side, then using CRLNet to detect the different fruit tree images, and finally, each detection box is then counted to obtain the production for each fruit tree. Although this study shows strong results on the peach detection task, the method in this study only takes into account the uncontrolled light problem in the orchard scene, and overexposure due to heavy rain, fog, and strong direct sunlight are common problems in the real working environment. When these extreme environments occur, the amount of information in the RGB image is drastically reduced, which leads to an increase in the imbalance between the information of the two modalities and, therefore, brings about a degradation in the detection performance of CRLNet. Subsequent research will proceed to design a reasonable image preprocessing network to regulate the amount of information in the captured image and optimize it at the input side, to solve the problem of peach recognition in extreme scenarios, and to further expand the practical value and application scenarios of CRLNet.

## 5. Conclusions

This study proposes a novel method for orchard peach recognition based on RGB and depth images. CRLNet, which accommodates multimodal image inputs, was designed by extending the state-of-the-art single-stream detector YOLOv9. To address the issue of uncontrolled lighting conditions in orchards, the Local–Global Enhanced Module (LGEM) was proposed, as well as filtering out interference from strong light and hollow noise—an aspect rarely considered in previous studies. This significantly improved the final detection accuracy. To fully utilize multimodal information, unique differential mode information was preserved and redundant common mode information suppressed. The Rough-Fine Fusion Module (RFAM) was embedded, which leverages cross-attention and coordinate attention mechanisms to precisely fuse multimodal features with significant differences, further enhancing the detection accuracy. During the experimental phase, CRLNet achieved an $mAP_{50}$ of 97.13% on the testing set, demonstrating the model’s effectiveness in peach detection. Additionally, ablation studies confirmed the efficacy of both the LGEM and RFAM, as well as their interaction. In the generalization experiments, CRLNet achieved an $mAP_{50}$ of 92.43% on a bagged peach dataset, proving the model’s robust generalization capability. Comparative experiments further showed that CRLNet outperformed other networks, effectively improving fruit recognition in orchard operations.

To further optimize the precise localization of fruits in complex scenarios, future research will continue to explore data preprocessing techniques, such as image brightness adjustment, to enhance the model’s robustness under uncontrolled lighting conditions. Additionally, future work should establish a large-scale fruit dataset and employ continual learning techniques to expand the model’s fruit detection capabilities, thereby increasing its practical value. Implementing these strategies will significantly improve the model’s stability and usability, providing more efficient technical support for automated pruning, thinning, and harvesting in fruit cultivation.

## Figures and Tables

**Table 1:** Experimental environment and training parameters for this study.

Hardware environment	CPU	22 vCPU AMD EPYC 7T83 64-Core Processor
	GPU	RTX 4090 × 2(24 GB) × 1
	RAM	30 GB
	Hard disk	System disk: 30 GB. Data disk: 50 GB
Software environment	Systems	Ubuntu20.04
	CUDA	11.8
	Pytorch	2.0
	Python	3.8
Parameters	Learning rate decay	0.0005
	Warm-up epoch	3
	Warm-up Lr	0.1
	Momentum	0.975
	Close mosaics epoch	30
	Lr	0.01
	Optimizer	Adam
	Epoch	200
	Batch size	32
	Size of input images	640 × 640

**Table 2:** Comparison of data from modal combination experiments, where rows 1, 2, and 3 denote RGB + RGB, Depth + Depth, and IR + IR, respectively. Bold indicates maximum value.

RGB	Depth	Inf	Module	${\mathrm{mAP}}_{50}$ (All)	${\mathrm{mAP}}_{50:95}$ (All)	${\mathrm{mAP}}_{50}$ (False)	${\mathrm{mAP}}_{50}$ (Leaf)	${\mathrm{mAP}}_{50}$ (Branch)	${\mathrm{mAP}}_{50}$ (Fruit)
✓				96.11%	62.51%	95.28%	96.05%	96.48%	**96.42%**
	✓			83.43%	45.33%	83.13%	83.52%	84.18%	82.90%
		✓		85.91%	47.82%	85.21%	85.34%	87.38%	85.69%
✓	✓			95.73%	62.01%	94.82%	95.59%	96.28%	96.21%
✓		✓		94.65%	61.50%	95.01%	94.69%	95.17%	93.63%
	✓	✓		85.24%	47.62%	83.81%	84.40%	86.68%	86.18%
✓		✓	✓	95.20%	61.14%	95.33%	95.07%	95.78%	94.62%
✓	✓		✓	**97.13%**	**69.44%**	**97.84%**	**98.63%**	**97.62%**	94.44%

**Table 3:** Performance analysis of the three-stream network. Bold indicates maximum value.

LGEM	RFAM	Data Type	${\mathrm{mAP}}_{50}$ (All)	${\mathrm{mAP}}_{50:95}$ (All)	${\mathrm{mAP}}_{50}$ (False)	${\mathrm{mAP}}_{50}$ (Leaf)	${\mathrm{mAP}}_{50}$ (Branch)	${\mathrm{mAP}}_{50}$ (Fruit)
		RGB + Depth + Ir	95.04%	61.41%	96.12%	95.03%	96.15%	92.87%
✓		RGB + Depth + Ir	**96.01%**	64.07%	95.59%	95.82%	96.88%	**95.73%**
	✓	RGB + Depth + Ir	94.71%	63.02%	**96.37%**	95.85%	**97.26%**	89.35%
✓	✓	RGB + Depth + Ir	95.76%	**65.54%**	96.21%	**96.62%**	95.70%	94.51%

**Table 4:** Comparison of the accuracy of Rough Fusion, Fine Fusion, and the RFAM module. Bold indicates optimal value.

Method	All		False		Leaf		Branch		Fruit		Params
	${\mathrm{mAP}}_{50}$	${\mathrm{mAP}}_{50:95}$	${\mathrm{mAP}}_{50}$	${\mathrm{mAP}}_{50:95}$	${\mathrm{mAP}}_{50}$	${\mathrm{mAP}}_{50:95}$	${\mathrm{mAP}}_{50}$	${\mathrm{mAP}}_{50:95}$	${\mathrm{mAP}}_{50}$	${\mathrm{mAP}}_{50:95}$	
NO	**95.73%**	62.01%	94.82%	69.66%	95.59%	62.46%	96.28%	58.05%	**96.21%**	57.89%	**72.43 M**
RAM	94.77%	60.49%	94.23%	68.71%	95.74%	61.28%	96.59%	56.31%	92.52%	55.69%	88.01 M
FAM	95.33%	61.40%	95.42%	69.44%	94.94%	56.74%	94.91%	56.74%	95.62%	57.24%	73.20 M
RFAM	94.84%	**64.03%**	**95.73%**	**69.90%**	**95.69%**	**63.22%**	**96.61%**	**60.74%**	94.89%	**59.83%**	89.04 M

**Table 5:** Comparison of the accuracy of Fine Fusion using different attention replacements. Bold indicates optimal value.

Method	All		False		Leaf		Branch		Fruit	
	${\mathrm{mAP}}_{50}$	${\mathrm{mAP}}_{50:95}$	${\mathrm{mAP}}_{50}$	${\mathrm{mAP}}_{50:95}$	${\mathrm{mAP}}_{50}$	${\mathrm{mAP}}_{50:95}$	${\mathrm{mAP}}_{50}$	${\mathrm{mAP}}_{50:95}$	${\mathrm{mAP}}_{50}$	${\mathrm{mAP}}_{50:95}$
NO	95.73%	62.01%	94.82%	69.66%	95.59%	62.46%	96.28%	58.05%	**96.21%**	57.89%
ResCBAM [39]	90.91%	55.12%	93.80%	63.71%	93.89%	56.80%	91.33%	51.09%	84.41%	48.62%
SE [40]	96.58%	66.48%	96.42%	73.07%	96.74%	66.32%	97.27%	64.00%	95.75%	62.18%
ECA [41]	96.50%	65.27%	96.61%	72.31%	96.39%	65.20%	97.45%	61.82%	95.63%	62.04%
Ours	**97.13%**	**69.44%**	**97.84%**	**75.73%**	**98.63%**	**72.94%**	**97.62%**	**65.63%**	94.44%	**63.29%**

**Table 6:** Comparison of the accuracy of image inputs with different resolutions. Bold indicates optimal value.

Image Size	${\mathrm{mAP}}_{50}$ (All)	${\mathrm{mAP}}_{50:95}$ (All)	${\mathrm{mAP}}_{50}$ (False)	${\mathrm{mAP}}_{50}$ (Leaf)	${\mathrm{mAP}}_{50}$ (Branch)	${\mathrm{mAP}}_{50}$ (Fruit)
320	86.30%	54.51%	92.42%	92.18%	88.30%	72.39%
480	94.92%	63.01%	96.92%	95.08%	95.34%	91.33%
640	**97.13%**	**69.44%**	**97.84%**	**98.63%**	**97.62%**	**94.44%**

**Table 7:** Comparison of the accuracy of different sizes of detectors. Bold indicates optimal value.

Modal	Scale	${\mathrm{mAP}}_{50}$ (All)	${\mathrm{mAP}}_{50:95}$ (All)	${\mathrm{mAP}}_{50}$ (False)	${\mathrm{mAP}}_{50}$ (Leaf)	${\mathrm{mAP}}_{50}$ (Branch)	${\mathrm{mAP}}_{50}$ (Fruit)	Params	Speed
RGB	0.25	94.92%	61.45%	95.22%	94.93%	95.45%	94.14%	**21.23 M**	**9.33 ms**
RGB	0.5	95.50%	62.33%	94.83%	88.51%	90.67%	90.21%	29.36 M	9.71 ms
RGB	1	96.11%	62.51%	95.28%	96.05%	96.48%	96.42%	60.12 M	13.42 ms
Depth	0.25	83.02%	45.01%	83.34%	83.33%	82.67%	82.91%	21.23 M	9.33 ms
Depth	0.5	83.04%	45.78%	83.91%	84.01%	81.60%	82.72%	29.36 M	9.71 ms
Depth	1	83.42%	45.81%	83.80%	84.45%	83.33%	82.01%	60.12 M	13.42 ms
RGB + Depth	0.25	94.51%	66.45%	**97.89%**	97.03%	92.34%	91.00%	27.01 M	16.21 ms
RGB + Depth	0.5	95.93%	68.33%	97.72%	97.81%	96.02%	92.11%	40.11 M	18.74 ms
RGB + Depth	1	**97.13%**	**69.44%**	97.84%	**98.63%**	**97.62%**	**94.44%**	90.03 M	31.11 ms

**Table 8:** Ablation study of the detection network on the peach dataset, where the dual-stream YOLOv9 is used as the baseline, and M1 and M2 are RGB + RGB and Depth + Depth, respectively. M3–M6 are the combination of RGB + Depth. Bold indicates optimal value.

	RGB	Depth	LGEM	RFAM	${\mathrm{mAP}}_{50}$ (All)	${\mathrm{mAP}}_{50:95}$ (All)	${\mathrm{mAP}}_{50}$ (False)	${\mathrm{mAP}}_{50}$ (Leaf)	${\mathrm{mAP}}_{50}$ (Branch)	${\mathrm{mAP}}_{50}$ (Fruit)
M1	✓				96.11%	62.51%	95.28%	96.05%	96.48%	**96.42%**
M2		✓			83.43%	45.33%	83.13%	83.52%	84.18%	82.90%
M3	✓	✓			95.73%	62.01%	94.82%	95.59%	96.28%	96.21%
M4	✓	✓	✓		94.81%	64.01%	95.44%	95.32%	95.37%	93.01%
M5	✓	✓		✓	94.84%	64.03%	95.73%	95.69%	96.61%	94.89%
M6	✓	✓	✓	✓	**97.13%**	**69.44%**	**97.84%**	**98.63%**	**97.62%**	94.44%

**Table 9:** Comparison between mainstream target detection algorithms, both unimodal and multimodal. Bold indicates optimal value.

Model	Datatype	${\mathrm{mAP}}_{50}$	${\mathrm{mAP}}_{50:95}$ (All)	${\mathrm{mAP}}_{50}$ (False)	${\mathrm{mAP}}_{50}$ (Leaf)	${\mathrm{mAP}}_{50}$ (Branch)	${\mathrm{mAP}}_{50}$ (Fruit)	Speed	Parameters
YOLOv3	RGB	94.48%	60.62%	94.50%	95.56%	93.04%	94.94%	**8.33 ms**	**10.37 M**
YOLOv3	Depth	82.89%	77.82%	83.81%	85.89%	83.04%	78.82%	8.33 ms	10.37 M
YOLOv3	Ir	82.20%	44.43%	81.60%	78.12%	85.11%	84.03%	8.33 ms	10.37 M
YOLOv5	RGB	96.02%	61.33%	96.01%	96.32%	96.89%	94.78%	10.28 ms	53.13 M
YOLOv5	Depth	82.33%	79.45%	82.84%	83.93%	81.54%	80.91%	10.82 ms	53.13 M
YOLOv5	Ir	84.67%	46.23%	82.73%	83.78%	84.80%	87.33%	10.82 ms	53.13 M
YOLOv6	RGB	96.01%	61.01%	95.93%	95.48%	96.53%	95.92%	10.94 ms	11.09 M
YOLOv6	Depth	74.33%	39.12%	72.45%	68.34%	51.53%	70.82%	10.94 ms	11.09 M
YOLOv6	Ir	76.41%	41.50%	76.22%	73.93%	73.72%	81.92%	10.94 ms	11.09 M
YOLOv8	RGB	95.18%	61.11%	95.83%	94.82%	95.13%	94.89%	9.81 ms	43.61 M
YOLOv8	Depth	84.30%	45.56%	80.92%	78.44%	77.31%	84.30%	9.81 ms	43.61 M
YOLOv8	Ir	85.78%	46.32%	85.71%	83.92%	86.22%	87.34%	9.81 ms	43.61 M
YOLOv9	RGB	96.11%	62.51%	95.32%	96.09%	96.47%	96.43%	13.42 ms	60.12 M
YOLOv9	Depth	83.44%	45.78%	83.82%	84.50%	83.31%	82.01%	13.42 ms	60.12 M
YOLOv9	Ir	85.89%	47.23%	84.44%	85.37%	88.32%	85.44%	13.42 ms	60.12 M
RTDETR	RGB	91.21%	57.77%	91.80%	90.45%	90.89%	91.42%	31.33 ms	41.94 M
RTDETR	Depth	83.29%	41.01%	82.03%	81.92%	90.01%	79.11%	31.33 ms	41.94 M
RTDETR	Ir	58.13%	29.52%	65.53%	58.72%	39.51%	68.82%	31.33 ms	41.94 M
C MASF	RGB + Depth	95.21%	61.19%	95.44%	95.43%	95.22%	94.89%	25.44 ms	72.57 M
AF M	RGB + Depth	95.02%	62.42%	95.45%	95.52%	96.13%	92.92%	29.40 ms	88.30 M
SCA	RGB + Depth	95.48%	64.11%	93.56%	95.33%	95.91%	71.78%	24.21 ms	75.72 M
MIA	RGB + Depth	95.67%	64.23%	92.71%	96.04%	96.03%	71.62%	24.82 ms	82.02 M
Ours	RGB + Depth	**97.13%**	**69.44%**	**97.84%**	**98.63%**	**97.62%**	**94.44%**	31.11 ms	90.03 M

**Table 10:** Comparison of the detection accuracy on the bagged peach dataset. Bold indicates optimal value.

Model	P	R	${\mathrm{mAP}}_{50}$	${\mathrm{mAP}}_{50:95}$
YOLOv9-RGBD	82.10%	81.02%	87.49%	60.89%
AFM	85.33%	83.21%	89.12%	64.67%
CMASF	82.44%	78.93%	86.03%	57.72%
MIA	81.18%	80.12%	86.31%	58.22%
SCA	80.73%	79.24%	85.89%	58.12%
Ours	**88.78%**	**86.92%**	**92.43%**	**70.02%**

## Data Availability

The data used in this article are publicly available and can be accessed at the article’s footnote section URLs.
